# Wissen, nicht Wahrheit: Was ist eigentlich (pädagogisches) Wissen?

**DOI:** 10.1007/s12054-021-00451-2

**Published:** 2022-01-05

**Authors:** Katharina Vogel

**Affiliations:** Georg-August-Universität Göttingen, Göttingen, Deutschland

**Keywords:** Wissen, Wahrheit, Konstruktion

## Abstract

Wissen ist keine absolute Wahrheit, sondern immer sozial determiniert. Auch pädagogisches Wissen ist eine eher unübersichtliche Ansammlung von unterschiedlichen „Wahrheiten“ – sie lassen sich aber sortieren.

In den letzten Monaten hat die Corona-Pandemie die Frage danach, wann wir etwas wirklich wissen können, immer wieder in die Nachrichten gebracht: Wissen und Wahrheit scheinen relative Begriffe zu sein, „alternative Fakten“ sind auch „Fakten“. Ist das so? Was ist Wissen? Und: Was ist denn eigentlich pädagogisches Wissen?

Wenn wir im Alltag von Wissen sprechen, meinen wir damit in der Regel ganz unterschiedliche Dinge: Wir wissen, wann der Biomüll abgeholt wird, oder wir wissen, wie man eine Deckenlampe anschließt; wir sagen: „Opa Bernd weiß ganz viel über den Krieg“ oder „Tante Gabi weiß alles über Verbrennungsmotoren“. Schon im Alltag wird deutlich, dass es unterschiedliche Formen von Wissen gibt: Wissen als das Erinnern eines Datums (Biomüll) oder einer Tätigkeit (Deckenlampe), Wissen als Sammlung biografischer Erfahrungen und Erlebnisse (Krieg) oder als Sammlung von Informationen (Verbrennungsmotoren). Auch klar wird, dass Wissen eigentlich kein umfassend belastbarer Begriff zu sein scheint: Dass wir theoretisch das Datum kennen, an dem der Biomüll abgeholt wird, hilft uns wenig, wenn wir es praktisch andauernd vergessen; dass wir theoretisch wissen, wie man eine Deckenlampe anschließt, ist kein Trost, wenn wir die Kabel verwechseln und einen Kurzschluss verursachen; das, was Opa Bernd über den Krieg weiß, passt vielleicht nicht zu dem, was Historiker_innen über den Krieg wissen, und es kann sein, dass Tante Gabi zwar die Technik von Verbrennungsmotoren sehr interessant findet, aber noch nie in direkten Kontakt mit einem Verbrennungsmotor gekommen ist.

## „Haben Sie Kinder?“

Die Pädagogik gehört zu den Bereichen der Gesellschaft, die unter solchen Formen von alltagsweltlich und alltagssprachlich verwässerten Wissens-Begriffen besonders leiden. So wird z. B. kinderlosen Pädagog_innen wie Erzieher_innen oder Lehrer_innen von Eltern im Streitfall gerne vorgeworfen, sie hätten doch eigentlich gar keine Ahnung, weil sie ja gar keine eigenen Kinder hätten – so, als sei die Alltagserfahrung von Eltern der von ausgebildeten Pädagog_innen im Zweifelsfall automatisch überlegen. „Erziehung“ und „Bildung“ sind einerseits wissenschaftlich und berufspraktisch aufgeladene Begriffe, schließlich kann man z. B. „Erziehungswissenschaft“ an einer Universität studieren oder ausgebildete „Erzieher_in“ werden – andererseits „passieren“ Erziehung und Bildung im weitesten Sinne praktisch immer und überall, zwischen Eltern und Kindern, im Museum, beim Fernsehschauen, in der großen Pause auf dem Schulhof oder beim Waldspaziergang. Es stellt sich die Frage, was Erziehungswissenschaftler_innen und Pädagog_innen wissen, was Nicht-Erziehungswissenschaftler_innen und Nicht-Pädagoginnen nicht wissen, und was „Wissen“ in diesem Fall eigentlich bedeutet.

## Erziehungswissenschaftliches/pädagogisches Wissen: Wichtige Unterschiede

Ausgehend von unserem „Erziehung-passiert-überall“-Beispiel besteht eine erste wichtige Differenz darin, das Wissen rund um Erziehung, Bildung, Lernen etc. seinen dominantesten Bezugskategorien zuzuordnen. So kann man zunächst zwischen den Kategorien „Alltagswissen“, „Professionswissen“ und „Wissenschaftliches Wissen“ unterscheiden (für das folgende: Vogel 2019, S. 34–39), um pädagogische Wissensbestände zu sortieren. Maßgebliche Unterscheidungsachsen sind hier die jeweiligen Nutzer_innen pädagogischen Wissens, die Funktion, die das Wissen im jeweiligen Fall erfüllt oder erfüllen soll, und die Form des Wissenserwerbs, also quasi die Bezugsquelle des Wissens, um das es geht.

## Pädagogisches Alltagswissen

Pädagogisches Alltagswissen wird von Laien, also z. B. Eltern, genutzt, um alltägliche Probleme zu lösen. Das Wissen zur Problembewältigung erhält man im Wesentlichen durch eigene Alltagserfahrungen und die alltagsweltliche Beschäftigung mit pädagogischen Themen, z. B. Laien-Ratgebern, Blogs oder Dokumentationen. Wenn z. B. Eltern ihren Kindern Medienzeiten zuteilen und ein Kind pro Tag nur eine Stunde lang fernsehen darf, bevor es im Garten spielen soll, ist das ein gutes Beispiel für pädagogisches Alltagswissen: Die Eltern treffen ihre Entscheidung auf Basis „irgendwie“ gewussten Wissens, z. B. weil sie die Erfahrung gemacht haben, dass ihr Kind unausgelastet ist, wenn es länger fernschaut, oder weil sie meinen, Fernsehen würde Kinder verblöden (= schlecht), wohingegen draußen Spielen gesund und natürlich sei (= gut). Alltagswissen ist also ein eher unzusammenhängendes Gebilde von Wissensbeständen und Handlungsregeln, die sowohl erfahrungsbasiert (vielleicht sogar: wissenschaftlich „getestet“!) als auch normativ geprägt sein können, und die sich in erster Linie im Alltag bewähren müssen.

## Pädagogisches Professionswissen

Pädagogisches Professionswissen unterscheidet sich von pädagogischem Alltagswissen zunächst dadurch, dass es nicht von Laien, sondern von pädagogischen Professionellen, also Pädagog_innen mit mehrjähriger Berufsausbildung, genutzt wird. Auch seine Funktion besteht darin, Probleme zu lösen, aber eben nicht die des privaten Alltags, sondern die des Berufsalltags. Dabei müssen sich die Probleme, die gelöst werden sollen, an sich gar nicht zwingend von denen des Alltags unterscheiden: Medienzeiten, Benehmen am Mittagstisch, Umgang mit anderen Kindern z. B. sind Probleme, die in beiden Welten vorkommen. Ihre Bearbeitung muss aber anderen Kriterien genügen. Während es im Falle des Alltagswissen genügt, wenn eine Lösung irgendwie funktioniert und sich für die Erziehenden bewährt, müssen Professionelle Lösungen finden, die den professionellen Standards ihrer jeweiligen Zunft entsprechen. Diese speisen sich wiederrum nicht nur, wie im Alltagswissen, aus alltäglichen Erfahrungen, sondern sind angereichert mit wissenschaftlichen Begründungen, rechtlichen Weisungen und berufspraktischem Erfahrungswissen. Während es z. B. „passieren“ kann, also: als normal gilt, wenn Eltern im Streit mit dem Kind herumbrüllen, um einen Konflikt augenscheinlich zu beenden, würde man von Erzieher_innen i. d. R. eine andere, nämlich: professionellere Problemlösungsstrategie zumindest erwarten.

Pädagogisches Professionswissen ist dadurch wesentlich weniger zufällig komponiert und gesättigt als Alltagswissen: Es besteht aus Wissen über das Handlungsfeld, in dem man sich bewegt, hält angemessene und erprobte Strategien für typische berufspraktische Probleme bereit und verfügt über Kriterien, die professionelles Handeln bewerten und im Zweifelsfall kritisieren können. Das ist für Professionelle unter anderem deshalb wichtig, weil sie „gegenüber ihrer Klientel stets damit rechnen müssen, dass ihr Handeln – gerade weil es hoch verantwortlich ist und tief in die Lebenspraxis von Menschen eingreifen muss – auf seine Begründung und Angemessenheit befragt werden kann“ (Helsper 2021, S. 135–136).

## Wissenschaftliches pädagogisches Wissen

Wissenschaftliches pädagogisches Wissen wird von Wissenschaftler_innen, aber auch von z. B. Politiker_innen, Bildungsplaner_innen und in Teilen auch von Angehörigen pädagogischer Professionen genutzt und versickert manchmal bis in die Welt des Alltagswissens. In der Medizin gibt es dafür gerade naheliegende Beispiele: Wissenschaftler_innen erzeugen wissenschaftliches Wissen über Covid-19; dieses Wissen wird von anderen Wissenschaftler_innen (weiter-)verwendet, aber eben auch von Politiker_innen genutzt, die auf Basis dieses Wissens Entscheidungen treffen. Diese Entscheidungen – und mit ihnen: ihr Wissen – diffundieren bis in die medizinische Berufspraxis, und, in diesem Fall, auch in die Alltagswelt, wenn auch in vereinfachter Form: Wir, die Laien, wissen z. B., dass man sich zum Schutz vor einer Übertragung des Virus die Hände waschen, Masken tragen und Abstand halten soll, vielleicht wissen wir auch rudimentär etwas über den Inzidenzbegriff – die ursprünglichen, komplexen wissenschaftlichen Studien, die sich mit dem Virus beschäftigen, und die im letzten Schritt zu diesen Handlungsempfehlungen führen, würden wir aber wohl nicht verstehen können.

Wissenschaftliches Wissen wird in den Medien der Wissenschaft erzeugt und entsteht in jeweils der Wissenschaft eigenen Theoriesprachen und Forschungssettings, es beruft sich auf methodologisch und methodisch abgesicherte Formen der Wissensproduktion. Von einer medizinischen Fallstudie über die Viruslast in Aerosolen bis zur Handlungsempfehlung „Maske tragen“ ist es dementsprechend ein langer Weg, und in den meisten Fällen schafft es wissenschaftliches Wissen gar nicht erst oder jedenfalls nicht unmittelbar in unsere Alltagswelt: Oder wussten Sie vor der Covid-19-Pandemie etwas über mRNA-Impfstoffe oder Inzidenzwerte?

## Grenzen von Wissen

Das Beispiel der Covid-19-Pandemie verweist auf zwei Grundprobleme, die die vorgestellten Wissenssorten mit sich bringen:

1. Der Transfer zwischen den Wissensformen ist aus unterschiedlichen Gründen nicht einfach, z. B., weil eine wissenschaftliche Erkenntnis sich nicht „einfach so“ in die Praxis übersetzen lässt.
2. Wissenschaftliches Wissen hat zwar den Anspruch, objektiv „richtige“ bzw. „wahre“ Urteile zu fällen, es will also nicht – wie z. B. die pädagogische Praxis – zwischen „gut/schlecht“, sondern zwischen „wahr/falsch“ unterscheiden können, aber das gelingt augenscheinlich nicht immer: auch in der Wissenschaft gibt es konkurrierende „Wahrheiten“, und nicht selten waren sich Virus-Expert_innen im letzten Jahr darüber uneinig, was stimmt und was nicht.

Das erste Problem führt uns zurück in die Welt der Pädagogik und erfordert eine weitere Differenz, die für pädagogisches Wissen wichtig ist, und die die Wissensformen, mit denen wir uns bisher beschäftigt haben, ergänzt: Die zwischen „Pädagogik“ und „Erziehungswissenschaft“ (Horn 1999).

**Figure 1:**
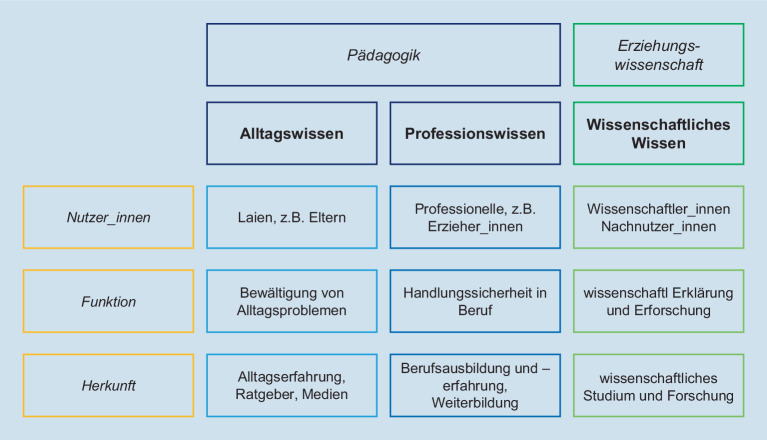

Unter „Pädagogik“ kann man Wissensbestände erfassen, „die das erziehungspraktische Handeln erzieherischer Akteure einerseits anleiten, mit denen die Akteure andererseits ihre Praxis reflektieren können“ (Horn 1999, S. 217). „Erziehungswissenschaft“ meint im Gegensatz dazu „die wissenschaftliche Beschäftigung mit allen Fragen der Erziehung und Bildung, die theoriebasiert und forschungsorientiert ist und nicht auf Erfahrungen aus der eigenen Erziehungspraxis beruht“ (Horn 1999, S. 218). Die Wissensformen „Alltagswissen“ und „Professionswissen“ lassen sich dementsprechend unter „Pädagogik“ zusammenfassen, die Form „Wissenschaftliches Wissen“ ist der Erziehungswissenschaft vorbehalten – und muss damit ganz anderen Gütekriterien genügen und ganz andere „Antworten“ liefern, als Alltags- oder Professionswissen das tun (s. Abb. 1).

## Was lernt man im wissenschaftlichen Studium?

Das führt zu einem Transferproblem pädagogischer und erziehungswissenschaftlicher Wissensformen, das insbesondere im Hochschulstudium deutlich wird: Weder die Studiengangstitel (z. B. „Diplom-Pädagogik“ oder „Bachelor of Arts Erziehungswissenschaft“) erzeugen ein eindeutiges Bild von dem, was Studierende erwartet, noch sind die Studierenden selbst sich darüber einig, was sie mit dem Studium bezwecken: Obwohl sich das Studium theoretisch ausschließlich in der Welt der Wissenschaft bewegt und dementsprechend erziehungswissenschaftliches, also eben nicht: pädagogisches, Wissen bereitstellen soll, begegnet man im Rahmen des Studiums i. d. R. beiden Wissensformen. Die Erwartung der meisten Studierenden ist, „etwas überspitzt formuliert: ‚Alles, was ich für die berufliche Praxis brauche, lerne ich im Studium‘ und ‚Alles, was im Studium an Theorien angeboten bzw. verlangt wird, muss auch berufsrelevant sein‘“ (Vogel 2019, S. 35). Ruft man sich die eben eingeführten Differenzen ins Gedächtnis, kann diese Erwartung nur enttäuscht werden, und der „wissenschaftlich ausgebildete Praktiker“ (Lüders 1987) bleibt eine merkwürdige Sonderfigur der wissenschaftlichen Pädagogik – oder eben: Erziehungswissenschaft.

## Erziehungswissenschaftliches Wissen löst keine pädagogischen Probleme

Auch, wenn es zunächst merkwürdig klingt: Erziehungswissenschaftliches Wissen ist nur sehr eingeschränkt dazu geeignet, pädagogische Fragen zu beantworten oder gar pädagogische Probleme zu lösen. Schulleistungsstudien wie die berühmt-berüchtigte PISA-Studie z. B. können – auf Basis komplexer Forschungen – zwar zeigen, wo, wann und inwiefern leistungsferne Faktoren in den Bildungskarrieren von Kindern und Jugendlichen eine Rolle spielen, z. B. indem sie messen, welchen Einfluss die soziale Herkunft auf den Bildungserfolg von Jugendlichen hat; sie können aber nicht direkt davon ableiten, ob ein Bildungssystem „gerecht“ oder „ungerecht“ ist oder wie Lehrer_innen in ihrer beruflichen Praxis mit Kindern aus bildungsfernen Haushalten umgehen sollten – das sind Fragen, die mit der PISA-Studie selbst nichts mehr zu tun haben, auch wenn die Studienergebnisse natürlich dazu genutzt werden können, diese Fragen zu bearbeiten.

Bleibt die Frage, ob und wenn ja wann Wissen „wahr“ ist und warum ausgerechnet wissenschaftliches Wissen, das den strengsten Wahrheitskriterien unterliegt, sich im letzten Jahr vermeintlich so oft als „falsch“ herausgestellt hat, weil es gleich mehrere „Wahrheiten“ gab: Gefühlt änderte sich beinahe täglich das „Wissen“ über Covid-19, Ansteckungsgefahren, die Wirksamkeit von Masken, Impfstoffen usw., und manchmal widersprachen sich wissenschaftliche Expert_innen gleich ganz grundsätzlich. Der Wissenschaft wurde vorgeworfen, eben doch nicht so überlegen und allwissend zu sein, wie sie sich – angeblich – selbst gerne sieht.

## Wissen im Wandel

Ein einfacher Grund für rückblickend „falsches“ Wissen besteht darin, dass Wissen nicht starr ist, sondern ständig neu erforscht, aus einem anderen Blickwinkel betrachtet, ergänzt und erneuert wird. Das gilt in der Virus-Forschung genauso wie in der Erziehungswissenschaft: Auch hier werden Phänomene der Erziehung und Bildung, z. B. Erfolg in der Schule, Fremdsprachenerwerb oder Demokratielernen immer wieder neu und immer wieder anders erforscht und diskutiert, neue Erkenntnisse aus den Nachbardisziplinen, z. B. der Psychologie, werden ergänzt und führen zu anderen Ergebnissen als früher. Der stetige Wandel von dem, was als „Wissen“ gilt, macht auch vor Alltags- und Professionswissen nicht Halt: „Wusste“ man z. B. in den 1950er-Jahren, dass Gewalt in der Erziehung dann und wann nützlich oder sogar notwendig ist, „wissen“ Eltern heute i. d. R., dass sie ihrem Kind damit ausschließlich Schaden zufügen; „wusste“ man im 18. Jahrhundert, dass Onanie jungen Knaben schadet, weil es den Körper schwächt und der Seele schadet, „weiß“ man heute, dass Onanie ein physisch und psychisch unproblematischer, „normaler“ Bestandteil von Sexualität ist.

## Auch „Fakten“ sind nicht immer „wahr“

Ist Wissen deshalb immer relativ und nie wirklich „wahr“? Ja und Nein. Dem Mediziner Ludwik Fleck, der später als Wissenschafts- und Erkenntnistheoretiker bekannt werden wird, fällt Anfang des 20. Jahrhunderts bei seinen medizinischen Studien etwas Merkwürdiges auf: Er beschäftigt sich mit Abbildungen von menschlichen Geschlechtsorganen in unterschiedlichen wissenschaftlichen Lehrbüchern und vergleicht verschiedene Darstellungen seit dem 17. Jahrhundert miteinander. Dabei findet er – obwohl er viele gute, detailreiche Bilder vor sich hat – „kein einziges naturgetreues“ (Fleck, [1935] 1980, S. 48), und das liegt nicht nur an den technischen Möglichkeiten der Visualisierung: Alle Bilder haben den Anspruch, die „Wirklichkeit“ abzubilden, und doch unterscheiden sie sich alle voneinander. Mal werden bestimmte Details anders betont oder angeordnet, mal wird etwas in den Vorder- oder Hintergrund gerückt. Alle Abbildungen sind „richtig“, aber keine ist wirklich „wahr“. Für Fleck wird diese Beobachtung einer von mehreren Gründen, sich mit der Frage zu beschäftigen, was eigentlich eine wissenschaftliche „Tatsache“ ist – und wie sie entsteht.

## Wahrheit ist nie relativ oder subjektiv

Fleck kommt zu dem Schluss, dass „Wahrheit“, so relativ sie auch wirken mag, nie relativ oder subjektiv ist. Sie ist aber, in seinen Worten, „vollständig determiniert“ (Fleck, [1935] 1980, S. 131). Wenn wir im Beispiel der Geschlechtsorgane bleiben, heißt das: Die Abbildungen der Geschlechtsorgane fallen nicht deshalb so unterschiedlich aus, weil manche „richtig/wahr“ und andere „unrichtig/falsch“ sind; sie fallen unterschiedlich aus, weil die Perspektive derjenigen, die die Abbildungen erstellen, so entscheidend (determinierend) für das ist, was abgebildet wird, dass die „Wahrheit“ unterschiedlich aussieht: Spezialist_innen für Geschlechtskrankheiten werden immer etwas anderes sehen (und abbilden), als Spezialist_innen für Fruchtbarkeitsfragen, und Geschlechterforscher_innen der Gegenwart würden vielleicht nicht mal die Unterscheidung „männlich“ und „weiblich“ vornehmen, die zu Flecks Zeiten noch uneingeschränkt vorherrschte.

## Wahrheit ist konstruiert – aber nicht willkürlich

Wahrheit ist deshalb nicht relativ oder subjektiv, aber sie hängt ab von den Sinnwelten derer, die sie erzeugen. Das funktioniert nicht willkürlich: Sie könnten also in die Abbildungen, die Fleck betrachtet, nicht einfach das Foto eines Feuerwehrautos legen und behaupten, für Sie sei das ab jetzt ein Penis. Die Orte, an denen Wahrheit „gemacht“ wird, an denen also Wissen erzeugt wird, nennt Fleck Denkkollektive. In ihnen herrschen bestimmte, ihre Mitglieder vereinende Ansichten, Vorannahmen, Weltbilder, kurz: Wahrheiten und Wissenssammlungen vor, die sie von anderen Denkkollektiven unterscheiden. Denkkollektive sind keine Vereine, denen man beitreten und die man wieder verlassen kann. Es sind eher sich selbstläufig verdichtende Weltbilder, deren Grenzen untereinander oft erst im Streitfall sichtbar werden, z. B. wenn Person X behauptet, die Welt sei eine Scheibe, und Person Y behauptet, sie sei rund – und beide sich sehr, sehr sicher sind, dass sie die Wahrheit sagen.

Flecks Theorie der „Entstehung und Entwicklung einer wissenschaftlichen Tatsache“ (1935/1980) wird für die Frage, was Wissen ist, deshalb so interessant, weil sie zeigt, dass auch das vermeintlich so sichere wissenschaftliche Wissen nicht wahr im absoluten Sinne ist, sondern immer das Ergebnis von komplexen, sozialen Konstruktionsleistungen – selbst in der Medizin, von der man meinen könnte, Biologie sei eben immer irgendwie Biologie, ganz ohne Interpretationsspielraum.

## Pädagogische / erziehungswissenschaftliche „Wahrheiten“

**Figure 2:**
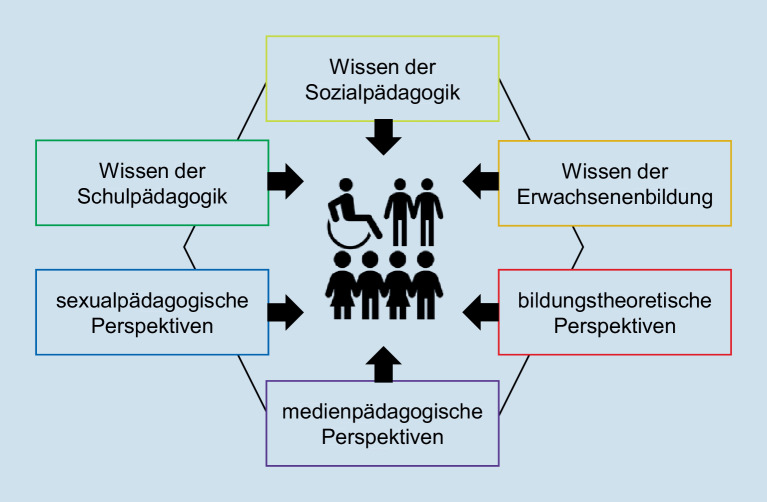

Was bedeutet das nun für pädagogisches oder erziehungswissenschaftliches Wissen? Wir können festhalten, dass unser Wissen abhängig ist von dem Feld, in dem wir uns bewegen: Praktiker_innen sind auf andere Wissensbestände angewiesen als Wissenschaftler_innen, obwohl sich ihre Gegenstandsbereiche und manchmal auch ihre Ausbildungswege durchaus überschneiden. Wissenschaftlich forschende Schulpädagog_innen werden, wenn sie mit Schüler_innen konfrontiert werden, andere Dinge „sehen“ und „wissen“ als wissenschaftlich forschende Sozialpädagog_innen, wenn sie dieselbe Schüler_innengruppe anschauen. Je nachdem, ob man zu den Berufspädagog_innen, Erwachsenenbildner_innen, Sozialpädagog_innen, Geschlechterforscher_innen oder den Schulpädagog_innen gehört – immer wird man, obwohl man sich augenscheinlich dem gleichen „Objekt“ zuwendet, etwas anderes für relevant halten, unwichtig finden oder neu entdecken, als die übrigen Beobachter_innen das tun. Für die einen sind psychologische Erkenntnisse wichtig, um ein bestimmtes Phänomen zu erklären, andere könnten auf die Soziologie nicht verzichten, um zu verstehen, was sie da eigentlich sehen (s. Abb. 2).

Es kann sein, dass psychologisch interessierte Schulpädagog_innen mit soziologisch denkenden Sozialpädagog_innen nicht besonders viel gemeinsam haben – auch wenn sich beide in der Pädagogik oder Erziehungswissenschaft bewegen. Sie werden aber – vielleicht! – im Großen und Ganzen mehr miteinander gemein haben, als mit schulpädagogisch interessierten Psycholog_innen oder sozialpädagogisch denkenden Soziolog_innen. Was jeweils erziehungswissenschaftliches Wissen oder nicht-erziehungswissenschaftliches Wissen ist, was pädagogisch relevant oder irrelevant ist, bleibt letztlich eine Frage der Perspektive.
